# High-resolution optical coherence tomography in pigmented choroidal lesions

**DOI:** 10.1186/s40942-025-00645-w

**Published:** 2025-02-27

**Authors:** Damian Jaggi, Shalin G. Aeberhard, Dmitri Artemiev, Martin S. Zinkernagel, Florian M. Heussen

**Affiliations:** 1 Department of Ophthalmology, Inselspital, Bern University Hospital, Freiburgstrasse 18, Bern, 3010 Switzerland; 2 Bern Photographic Reading Center, Inselspital, Bern University Hospital, Bern, Switzerland

**Keywords:** Pigmented lesion, Oct

## Abstract

**Purpose:**

Evaluating High-Resolution Optical Coherence Tomography (HR-OCT) versus standard spectral domain OCT in pigmented choroidal lesions.

**Methods:**

We screened a total of 40 subjects with pigmented choroidal lesions. Lesions were imaged on HR-OCT and standard OCT devices with and without enhanced depth imaging (EDI) mode. Images were graded for qualitative and quantitative aspects, like choriocapillaris compression and width, choroidal reflectivity and thickness, amongst others.

**Results:**

32 of the 40 subjects could be included in the image analysis, the rest were excluded due to insufficient imaging. HR-OCT EDI mode allowed visualization of the posterior choroidal extent in 11 lesions (11/32 = 34.4%) versus in six (6/32; 18.8%), four (4/30; 13.3%), and five cases (5/30; 16.7%) in normal HR-OCT (*n* = 32), standard OCT (*n* = 30) and OCT EDI mode (*n* = 30), respectively, albeit not significantly different. Choriocapillaris compression was evident in 10 cases on HR-OCT and equally visible in all imaging modes. Mean choriocapillaris thickness ranged from 11.6 to 13.9 microns (SD range 3.84–4.33), and compressed choriocapillaris thickness similarly ranged from a mean of 7.1 to 7.8 microns (SD range 2.20–3.55). Image quality declined significantly towards the periphery in three out of four modes (*p* = 0.0077 to *p* = 0.29).

**Conclusions:**

HR-OCT may provide better visibility of retinal and choroidal structures in pigmented choroidal lesions, although image quality is reduced in attempting to image peripheral lesions.

**Translational relevance:**

The prototype HR-OCT offers insights into clinical features of pigmented choroidal lesions that are not apparent on conventional OCT imaging. This supports the development of HR-OCTolution OCT devices.

## Introduction

Recent advancements in Optical Coherence Tomography (OCT) technology have significantly improved our ability to examine retinal structures, enhancing understanding of various retinopathies. High-Resolution OCT (HR-OCT) has shown promise in providing greater detail compared to conventional OCT systems, even at the cellular and subcellular levels [1, 2]. Some reports have demonstrated that HR-OCT may indeed be useful in pathology such as macular degeneration, diabetes or ocular surface lesions. For example, grading of known retinal biomarkers seemed to be more accurate and reliable on HR-OCT compared to standard OCT, and there is tentative evidence that HR-OCT may aid in making the distinction between malignant or benign ocular surface lesions [3–6]. Its effectiveness in the context of choroidal pathology, however, remains unknown.

Capturing pigmented choroidal lesions using OCT presents challenges, with issues such as signal loss towards the posterior margin of the lesion or the lesion’s size or peripheral location making it difficult to capture within the OCT scan pattern [7]. While HR-OCT OCT may not solve all these challenges, it can improve the visible resolution of certain structures, like the choriocapillaris. Better visualization may lead to a better understanding of the mechanisms underlying these choroidal lesions. For example, it has recently been suggested that the presence of hyperreflective rings on OCT angiography may be associated with a higher risk of malignancy [8]. Similarly, subretinal fluid imaging has shown that the optical density ratio of subretinal fluid may be associated with the potential for malignant transformation [9, 10].

This study aims to explore the capabilities of HR-OCT in evaluating pigmented choroidal lesions, employing comparative imaging with a conventional SPECTRALIS HRA + OCT device to determine any incremental details HR-OCT may reveal. We hypothesize that the superior axial resolution of HR-OCT (3 vs. 7 μm) may either reveal newly gradable features of choroidal lesions or, at least, provide better visibility of already known biomarkers and quantitative parameters.

## Methods

### Study design

**Fig. 1:**
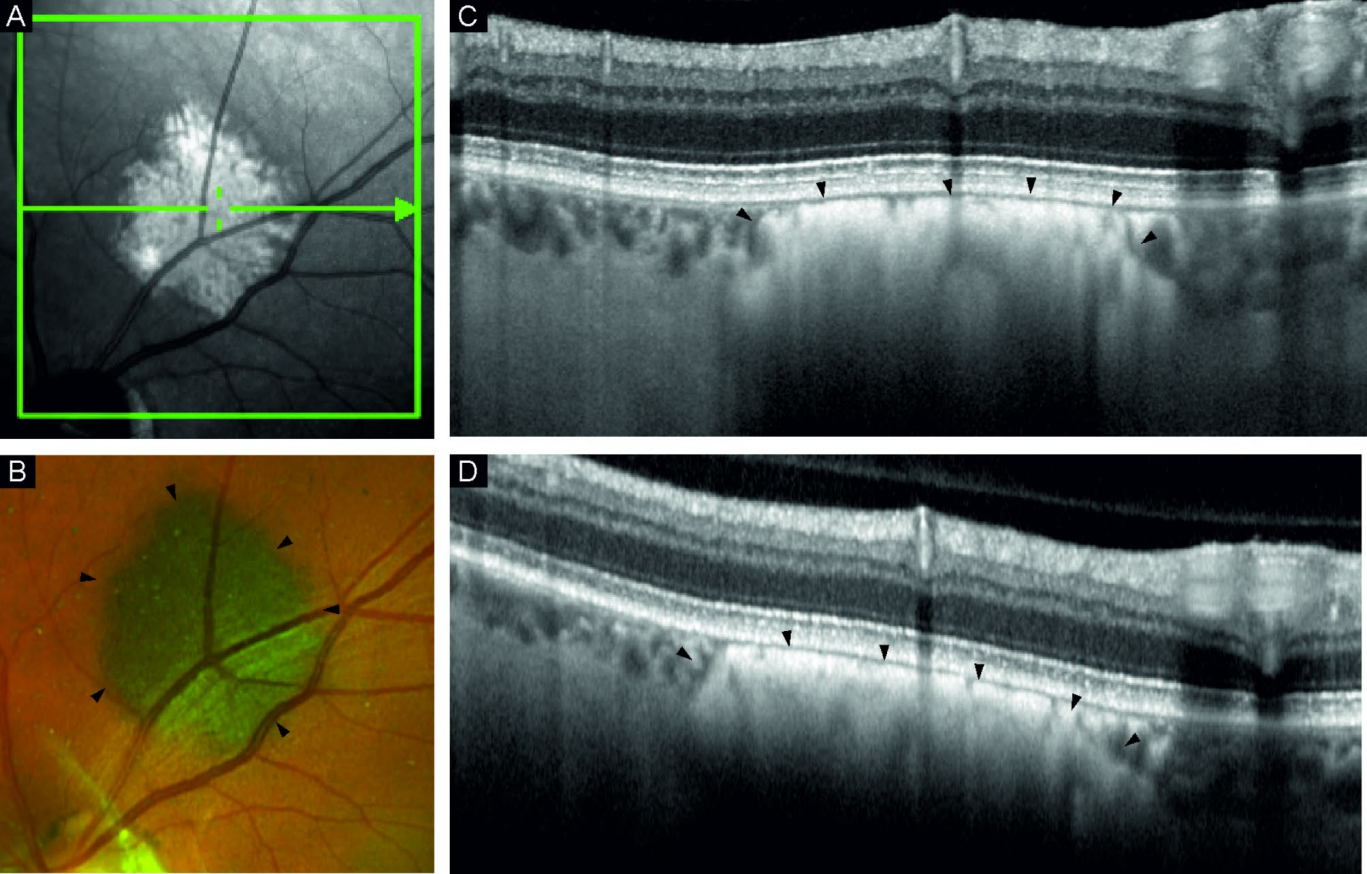
Multimodal imaging, with depiction of the lesion (black arrowheads). **A** shows an infrared image of the pigmented lesion in a left eye, superior to the optic disc. **B** shows a color image of the same lesion. **C** shows the high-resolution OCT B-scan through the lesion. **D** shows the conventional OCT B-scan through the lesion

This study enrolled a cohort of patients from specialty clinics at the Department of Ophthalmology, Inselspital - University Hospital Bern, from November until December 2023, each clinically diagnosed with a pigmented choroidal lesion. Participants gave written informed consent to participate in the study. The study was conducted in accordance with the Swiss Human Research Act, the ICH guidelines of Good Clinical Practice, the Declaration of Helsinki and was authorized by the local ethics committee (Kantonale Ethikkomission Bern, KEK, BASEC-ID. 2021-D0038). Participants underwent comprehensive OCT imaging employing both the prototype SPECTRALIS HR- OCT device and the standard SPECTRALIS spectral domain OCT (both from Heidelberg Engineering, Heidelberg, Germany). The technical specifications of the HR-OCT in our department have been described previously [1], notably the axial resolution is increased to about 3 microns compared to 7 microns on the standard platform. The imaging protocol remained consistent across both devices, incorporating volume scans that averaged 25 scans per B-Scan, a 60-micron spacing between adjacent B-scans within a volume scan, and a variable scan area depending on the size of the lesion. Lesions smaller than a 30-degree field of view were entirely captured within a single volume scan, while attempts were made to capture larger lesions as comprehensively as possible, ensuring inclusion of adjacent normal retina and choroid in the imaging (Fig. 1). Since there was a large number of scans acquired in each case, we opted to go with the smallest possible scan pattern size still capturing the lesions as described above. This helped to ensure patient comfort but also reliable image quality between the different scans. Enhanced depth imaging mode (EDI) was also utilized for each lesion, with both activation and deactivation on each device. EDI is a software feature for SPECTRALIS OCT that allows better visibility of choroidal structures [11]. Macular scans as well as scans over the choroidal lesions were acquired. Supplementary imaging included color fundus and autofluorescence imaging via the Optos California device (Optos plc, Dunfermline, UK) for all participants.

### Grading

**Fig. 2:**
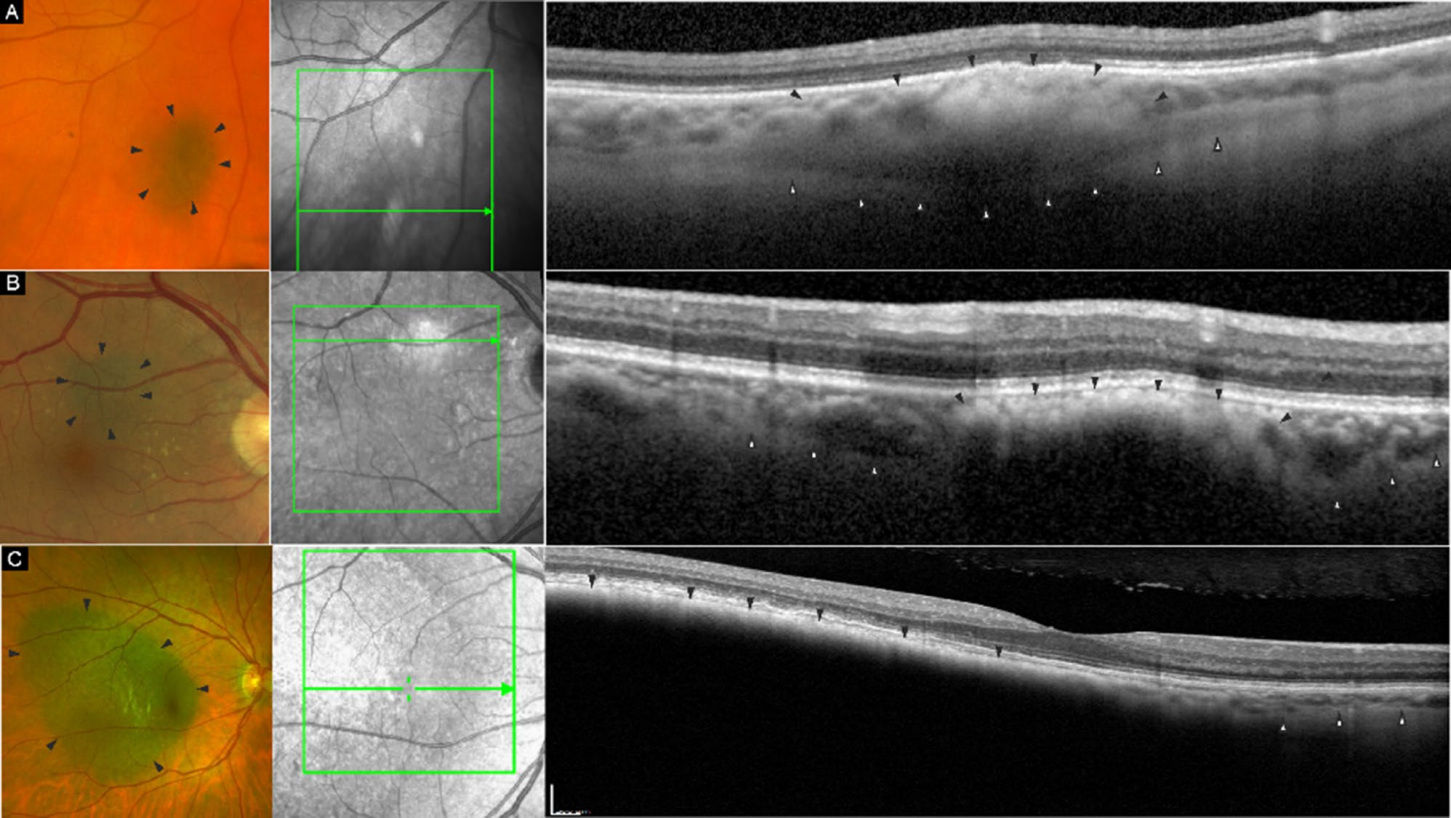
Detection of the posterior border. The upper grouped images (**A**) show a color fundus image, an infrared image and a corresponding high-resolution OCT B-scan, with depicted lesion boarders (black arrowheads). Detection of the posterior border was possible where reflectivity changed from hypo- to hyperrflectivity (white arrowheads). The middle grouped images (**B**) show the corresponding modalities of a lesion, where the choroids posterior border could only be captured in the normal tissue adjacent to the lesion (white arrowheads). The lower grouped images (**C**), show the corresponding imaging modalities of a larger lesion, where the posterior choroidal border was only detectable in adjacent normal tissue (white arrowheads), but not in the lesion

Two retina specialists (DJ, FH) conducted an evaluation of the images, aiming to delineate structural details. The analysis encompassed specific features such as the integrity of retinal layers overlaying the choroidal lesion, the presence of sub-retinal or intra-retinal fluid (SRF/IRF), and the surface configuration of the lesion (flat, excavated, dome-shaped). Additionally, the visibility and, if feasible, the thickness of the lesion’s posterior margin were documented, utilizing the caliper tools of the Heyex imaging platform (Heidelberg Engineering, Heidelberg, Germany). The choriocapillaris (CC) was assessed over the choroidal lesion and in the adjacent normal choroid, noting any signs of choriocapillaris compression and measuring its thickness in both locations. For all thickness measurements (choroidal lesion and CC) the graders chose a representative B-Scan with the largest thickness of the structure in question within the OCT volume scan at their own discretion and used the SPECTRALIS in-built caliper tool. The graders also evaluated whether the internal reflectivity of the choroidal lesion was distinct enough to discern internal vasculature, albeit only at the superficial aspect of the lesion (Fig. 2). In case of disagreement, adjudication took place in the presence of a third grader (MZ) and a final grading was agreed upon. Finally, OCT image quality was noted (index value provided by the Heyex software) and distance from both the center of the optic nerve head (ONH) and the fovea to the closest margin of the lesion was measured on Optomap images. The anatomic location of the fovea on the Optomap images was confirmed by cross-referencing macular OCT scans in all patients.

### Statistical analysis

Descriptive statistics were used to evaluate the features observed in the OCT images. Comparative analyses between the HR-OCT and conventional OCT images were performed using Bland Altman plots for numeric parameters. To detect differences in the visibility of retinal and choroidal structures with a binary grading scale, Kruskal-Wallis test with Dunns correction for multiple comparisons was made. Image Quality and distances to the fovea and optic nerve head passed all available tests for normality and were then correlated using Pearson correlations.

## Results

Out of 40 patients screened, 32 (12 female, 37.5%) had choroidal lesions that were within reach for imaging of acceptable quality, eight lesions were too peripheral. Mean age was 69.04 (SD ± 8.9, Range 40–82) years. All patients had only one eye involved (13 right, 19 left). Eleven had clear signs of drusen on the location of the lesion. Twenty-six lesions were diagnosed as ”nevi”, while 6 were described as “indeterminate”. Indeterminate lesions were defined as choroidal pigmented lesions with at least two significant risk factors for potential malignancy, i.e. thickness > 2 mm, orange pigment, symptoms and other factors). An ocular oncology specialist assigned diagnosis of indeterminate lesion in the respective subspecialty clinic. Out of the 32 cases, two (6.25%) had HR-OCT but not standard OCT scans taken due to a protocol error. This is indicated for the respective statistical tests where applicable.

Comparing the different imaging modalities, HR-OCT generally provided better visualization of retinal and choroidal layers, compared to the standard OCT (Fig. 1). HR-OCT EDI mode allowed visualization of the posterior border in 11 lesions (11/32 = 34.4%) versus in six (6/32; 18.8%), four (4/30; 13.3%), and five cases (5/30; 16.7%) in normal HR-OCT (*n* = 32), standard OCT (*n* = 30) and OCT EDI mode (*n* = 30), respectively. A summary of the values are provided in Table 1. Statistical analyses did not show significant differences between the graded images. Choriocapillaris compression was evident in 10 cases on HR-OCT and equally visible in all imaging modes (*p* > 0.05). Mean choriocapillaris thickness ranged from 11.6 to 13.9 μm (SD range 3.84–4.33 μm) between the imaging modes, and compressed choriocapillaris thickness similarly ranged from a mean of 7.1 to 7.8 μm (SD range 2.20–3.55 μm), again, with no statistically significant difference.

**Table 1:** Comparison of four different OCT modalities; high-resolution OCT (HR-OCT), OCT, high-resolution enhanced depth of imaging (hr-EDI-OCT), enhanced depth of imaging OCT (EDI-OCT)

	HR OCT	OCT	HR EDI OCT	EDI OCT
Retinal Disruption	14	15	14	15
Subretinal Fluid	6	6	6	6
Choriocapillaris Compression	7	10	7	9
Cell/pigment migration	6	2**	6	2**
Bruch membrane visible and disrupted	3	0	5	0
Choriocapillaris Width Mean (SD)	*n* = 25* 11.00 (3.42)	*n* = 25 12.76 (4.01)	*n* = 24 11.96 (3.90)	*n* = 26 13.58 (3.74)
Choriocapillaris Compression Width	*n* = 6* 7.5 (2.07)	*n* = 9 7.56 (3.17)	*n* = 7 8.14 (1.77)	*n* = 10 7.6 (3.66)
Choroidal Thickness	*n* = 6* 409 (306.8)	*n* = 4 502 (374.2)	*n* = 11 416 (244.2)	*n* = 5 562 (345.6)

*Analysis only includes cases where the measurement of the respective feature was possible. **suspicion of cell/pigment migration but no clear confirmation

**Fig. 3:**
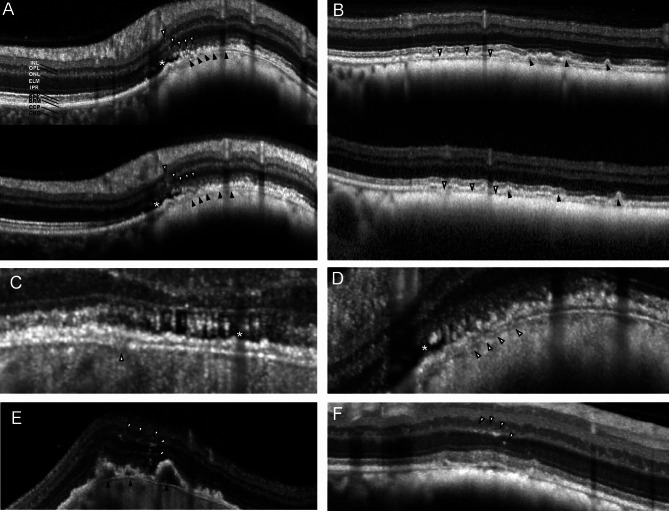
Selected Images depicting structures with better visibility on high-resolution OCT (HR-OCT) compared with standard spectral-domain OCT. **A** shows pigment or cell migration (white arrowheads) through the outer retina with better visibility on the HR-OCT (upper image). Also, Bruch’s membrane and its focal discontinuity is better distinguishable on the HR-OCT (black arrowheads). Subretinal fluid is present (asterisk). **B** presents a better visibility of the compressed choriocapillaris (white arrowheads) in the HR-OCT (upper image) than on conventional OCT (lower image). Drusen are present (black arrowheads). **C** and **D** show cases with local Bruch layer disruption (white arrowhead) and faint subretinal fluid (asterisk). **E** shows a case with pigment migration (white arrowheads) and drusen (black arrowheads). **F** shows a case with pigment migration (white arrowheads). INL, Inner nuclear layer. OPL, outer plexiform layer. ONL, outer nuclear layer. ELM, external limiting membrane. IPR, inner photoreceptor segments. OPR, outer photoreceptor segments. RPE, retinal pigment epithelium. BRM, Bruch membrane. CCP, choriocapillaris. CHO, choroid

Qualitatively, HR-OCT revealed new aspects that were not detectable on the standard OCT, such as cell or pigment migration in the outer retinal layers, or the choriocapillaris compression and focal Bruch’s membrane discontinuation over certain lesions (Fig. 3).

**Fig. 4:**
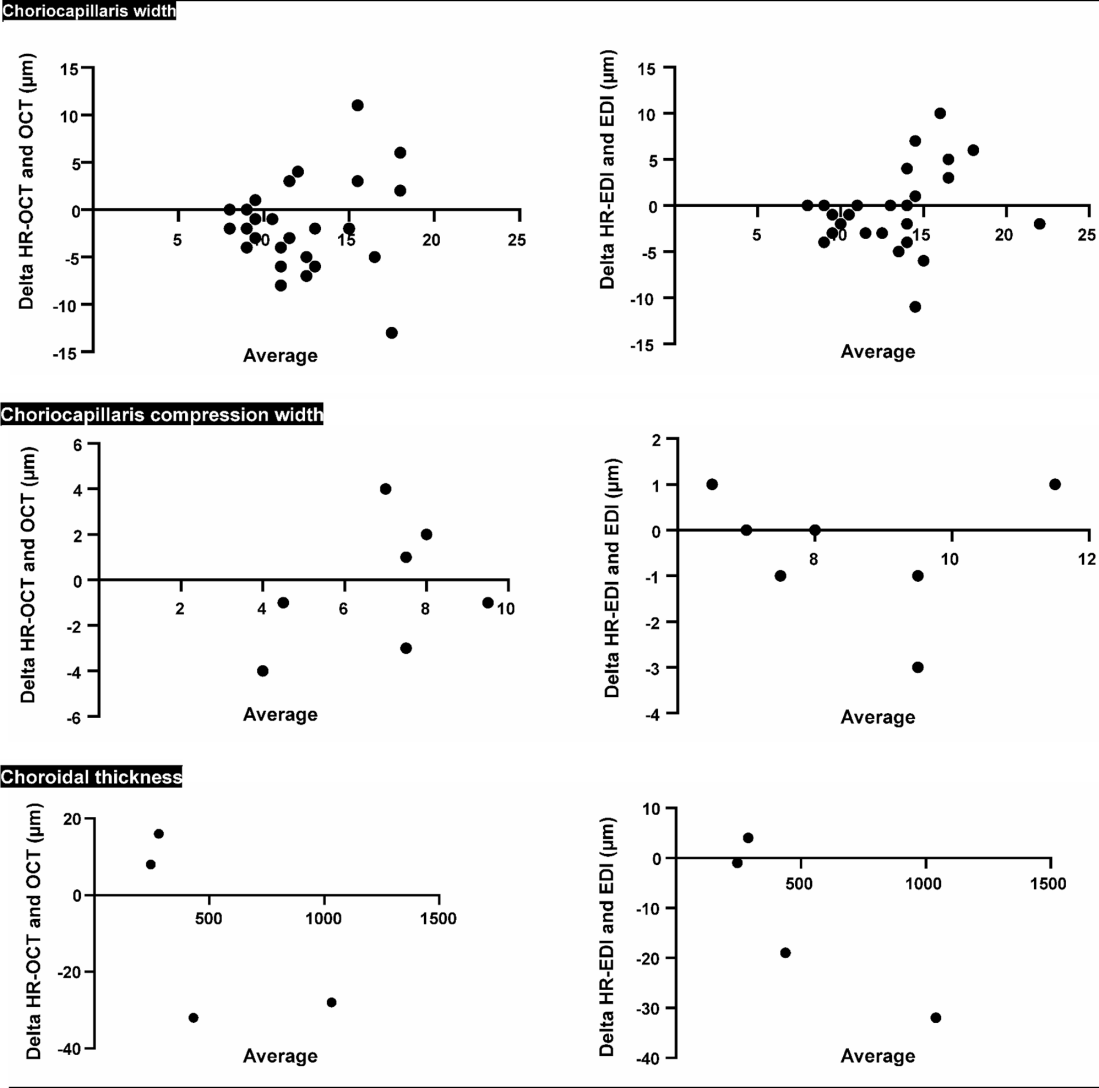
Bland Altman plots showing comparisons between the high-resolution OCT (HR-OCT) and the conventional OCT (left graphs), and between the high-resolution EDI mode (hr-EDI) and conventional EDI mode (right graphs). Comparisons were made for choriocapillaris width, choriocapillaris compression width, and choroidal thickness

Standard Bland Altman analysis confirmed no systematic over- or underestimation when comparing the different modalities between HR-OCT/OCT and HR-EDI /EDI, with bias values (all in µm) of -1.54 (SD 4.8) and − 0.75 (SD 4.4), -0.29 (SD 2.8) and − 0.43 (SD 1.4), -9.0 (SD 24.5) and − 12.0 (SD 16.6), for choriocapillaris width, choriocapillaris compression width, and choroidal thickness, respectively. In terms of choriocapillaris width measurements, greater average width led to more fluctuation between the modalities (Fig. 4).

Intergrader assesments are presented in Table 2. Generally, good intergrader reliability was abserved. HR-OCT showed slightly better intergrader reliability than standard OCT.

**Table 2:** Intergrader reliability for each parameter, measured with Cohen kappa for nominal values, and pearson correlation for choriocapillaris width. Choriocapillaris compression width and choroidal thickness are not included due to the small case numbers

	HR OCT	OCT	HR EDI OCT	EDI OCT
Retinal Disruption, cohen kappa, (disagreed cases)	0.75 (4)	0.688 (5)	0.75 (4)	0.688 (5)
Subretinal Fluid, cohen kappa, (disagreed cases)	1.0 (0)	1.0 (0)	1.0 (0)	1.0 (0)
Choriocapillaris Compression, cohen kappa, (disagreed cases)	0.626 (6)	0.51 (10)	0.64 (5)	0.56 (7)
Choriocapillaris Width Mean (SD), pearson r	0.84	0.65	0.74	0.65

The signal quality of the OCT scans on both devices declined towards the periphery, if the center was defined as the fovea (Pearson r^2^ = 0.22; *p* = 0.0077, r^2^ = 0.13; *p* = 0.04, r^2^ = 0.14; *p* = 0.03, r^2^ = 0.04; *p* = 0.29 ns., for HR-OCT, OCT, HR-EDI, EDI, respectively).

## Discussion

This study demonstrates the potential benefits and inherent limitations of HR-OCT in evaluating pigmented choroidal lesions. The choriocapillaris was one of the key structures we focused our analysis on, as we theorized that the superior axial resolution of HR-OCT at this tissue level might reveal greater differences to standard OCT. While quantitative thickness measurements of the choriocapillaris did not significantly differ between HR-OCT and standard OCT, HR-OCT did, in fact, provide superior visualization of both normal and compressed choriocapillaris. This may be of interest as these different presentations of the choriocapillaris may play a role in differentiating benign from malignant choroidal mass lesions. An analysis by Yu et al. suggested a correlation between choriocapillaris compression and subretinal fluid in a series of 3431 choroidal naevi [12]. Interestingly, the compression was also seen in smaller lesion although larger size was associated with more frequent choriocapillaris compression. The association of compression with subretinal fluid was true for incidence of new fluid as well as worsening of fluid levels over time. Investigating whether other changes in the choriocapillaris precede detectable changes in the overlying retinal layers could be similarly valuable for future research. We believe the superior axial resolution of HR-OCT may be of particular advantage in this situation.

Beyond the choriocapillaris, HR-OCT revealed additional structural details in some cases, such as suspected cell migration, that were missed on standard OCT. Macrophage Migration Inhibitory Factor (MIF) plays a role in the carcinogenesis of many cancer types, including uveal melanoma, and its inhibition can lead to cell migration [13, 14]. Thus, while the precise significance of our OCT findings remains unclear due to the lack of histologic confirmation, they may represent early signs of lesion reorganization or transformation.

Contrary to our qualitative assessment, we did not find any quantifiable differences in the measurements performed by the two OCT devices. Therefore, many of the clinical advantages of HR-OCT are qualitative in nature and difficult to support with objective measurements. A notable exception was the improved visualization of the posterior lesion margin with the HR-OCT EDI mode. Monitoring changes in this margin over time could serve as an early biomarker for lesion growth or regression. Especially in small, pigmented choroidal lesions, OCT-based thickness measurements are potentially more reliable than ultrasound measurements [15].

The study also highlights the key challenges facing HR-OCT. In line with previous research, our study confirms that while HR-OCT provides unparalleled central resolution, it suffers from rapid degradation of image quality towards the retinal periphery, much like other OCT devices. This proved challenging for imaging peripheral choroidal lesions comprehensively. Shah et al. reported, that only close to 50% of nevi in their series qualified for comprehensive OCT image review due difficulties with image acquisition [7].

The HR-OCT ability to image subcellular structures, as reported before [1], could not be replicated with certainty in the current series with eyes that have pathology and lesions outside the posterior pole. Although no simple solutions exist currently, advancements like computational image enhancement may help address these limitations in the future. Additionally, the lack of histologic validation makes definitive interpretation of cellular HR-OCT findings in diseased eyes problematic. Still, while it does not rival the resolution of an adaptive optics system, HR-OCT delivered the best in-vivo resolution of pigmented choroidal lesions over an area of 6 × 6 mm or more, yet [16].

In conclusion, HR-OCT may provide advantages over standard OCT when examining centrally located pigmented choroidal lesions. Future research focusing on peripheral image enhancement, standardized protocols, and correlation with clinical outcomes will be crucial to determine its place as a diagnostic and monitoring tool in ophthalmology.

## Data Availability

Data is provided within the manuscript or supplementary information files.

## Abbreviations

BCVA: Best corrected visual acuity
CI: Confidence interval
EDI: Enhanced depth imaging
OCT: Optical coherence tomography
HR-OCT: High resolution optical coherence tomography
SD: Standard deviation
